# Planned Peri-Extubation Fasting in Critically Ill Children: An International Survey of Practice

**DOI:** 10.3389/fped.2022.905058

**Published:** 2022-05-11

**Authors:** Tomasz Nabialek, Lyvonne N. Tume, Eloise Cercueil, Claire Morice, Lionel Bouvet, Florent Baudin, Frederic V. Valla

**Affiliations:** ^1^Pediatric Intensive Care, Royal Children’s Hospital, Melbourne, VIC, Australia; ^2^School of Health and Society, University of Salford, Manchester, United Kingdom; ^3^Pediatric Intensive Care, Lyon University Children Hospital, Hospices Civils de Lyon, Lyon, France; ^4^Department of Anesthesiology and Intensive Care, Lyon University Children Hospital, Hospices Civils de Lyon, Lyon, France

**Keywords:** pediatric intensive care, enteral nutrition, mechanical ventilation, aspiration, ventilator associated pneumonia, vomiting, energy deficit, protein deficit

## Abstract

**Introduction:**

Cumulative energy/protein deficit is associated with impaired outcomes in pediatric intensive care Units (PICU). Enteral nutrition is the preferred mode, but its delivery may be compromised by periods of feeding interruptions around procedures, with peri-extubation fasting the most common procedure. Currently, there is no evidence to guide the duration of the peri-extubation fasting in PICU. Therefore, we aimed to explore current PICU fasting practices around the time of extubation and the rationales supporting them.

**Materials and Methods:**

A cross sectional electronic survey was disseminated *via* the European Pediatric Intensive Care Society (ESPNIC) membership. Experienced senior nurses, dieticians or doctors were invited to complete the survey on behalf of their unit, and to describe their practice on PICU fasting prior to and after extubation.

**Results:**

We received responses from 122 PICUs internationally, mostly from Europe. The survey confirmed that fasting practices are often extrapolated from guidelines for fasting prior to elective anesthesia. However, there were striking differences in the duration of fasting times, with some units not fasting at all (in patients considered to be low risk), while others withheld feeding for all patients. Fasting following extubation also showed large variations in practice: 46 (38%) and 26 (21%) of PICUs withheld oral and gastric/jejunal nutrition more than 5 h, respectively, and 45 (37%) started oral feeding based on child demand. The risk of vomiting/aspiration and reducing nutritional deficit were the main reasons for fasting children [78 (64%)] or reducing fasting times [57 (47%)] respectively.

**Discussion:**

This variability in practices suggests that shorter fasting times might be safe. Shortening the duration of unnecessary fasting, as well as accelerating the extubation process could potentially be achieved by using other methods of assessing gastric emptiness, such as gastric point of care ultrasonography (POCUS). Yet only half of the units were aware of this technique, and very few used it.

## Introduction

Enteral nutrition (EN) is the recommended first line nutritional support in critically ill children (1, 2). One of the challenges for ensuring optimal EN delivery in the critically ill is the practice of feeding interruptions and fasting for procedures which may lead to energy and/or protein deficits and their related impaired outcomes (3, 4). Fasting for procedures is one of the most common reasons for EN interruptions in the pediatric intensive care unit (PICU) (5–7), with planned extubation being the most common procedure and most frequent single cause for fasting (5–7).

Guidance exists for recommended fasting times prior to planned anesthesia and sedation and has been updated recently, shortening fasting times, considering the risk of hypoglycemia, hypovolemia, and stress on children (8, 9). We anticipated that in view of the lack of specific recommendations for peri-extubation fasting of PICU patients, these practices may be influenced by the guidance for planned anesthesia, potentially exposing patients to unnecessary or prolonged periods of interrupted nutrition and potentially delaying extubation. Optimizing fasting practices prior to extubation may improve the nutritional support and accelerate the time to extubation. Therefore, we wanted to describe the current practices and rationales around fasting times prior to and after planned extubation (termed peri-extubation) in PICUs internationally.

## Materials and Methods

A cross sectional electronic survey design using the SurveyMonkey™ (Momentive) platform was undertaken. A 16-item mixed question type survey (in English) was developed by an expert team (four pediatric intensivists, two pediatric anesthesiologists working in PICU, and one senior PICU nurse) after reviewing the literature (Electronical Supplementary Material). After development, the survey was piloted on five relevant health care professionals (two pediatric intensivists, one pediatric anesthesiologist working in PICU, one senior PICU nurse and one PICU dietician) to check clarity and establish face validity. The survey introduction made it clear that this focused only on peri-extubation fasting practices of children aged 0–17 years (but not premature < 37 weeks gestation age infants) on PICUs. Fasting was defined as the planned interruption of enteral nutrition in anticipation of extubation. The survey explored PICU practices on (i) fasting times prior to extubation, (ii) gastric feeding tube aspiration practice, (iii) fasting times post extubation, (iv) the rationale for these fasting times (v) clinicians’ estimated rates of extubation failure and non-invasive ventilation use post extubation, and (vi) the use of gastric point of care ultra-sounding (POCUS) to assess gastric volume in this setting.

The survey link was emailed through our Pediatric Critical Care Nutrition Network and *via* professional society (ESPNIC) contacts in January 2022. Three reminders were sent a week apart to maximize response rates. Experienced senior nurses, dieticians or doctors were invited to complete the survey on behalf of their unit, with only a single response requested per center. If there was more than one response from a center, the first response was used for analysis. The study is reported according to the CHERRIES reporting checklist for e-surveys (10).

## Analysis

The survey responses were downloaded in a CVS file to Microsoft Excel for descriptive analysis. No inferential data analysis was planned, and data are presented as percentages and categories only, consistent with the study aim. Surveys were included even if they had some incomplete items and because of this, for each question we report how many respondents omitted the questions (both as number and percentage). Ethical approval was obtained from the Ethics Committee of the French Group for Pediatric Hepatology, Gastroenterology and Nutrition (30 November 2021, reference number 2021-036) and written consent was waived (Participation in the survey implied responders’ consent that their responses could be used for research, as written in the invitation letter of the survey).

## Results

### Respondent Characteristics

**FIGURE 1:**
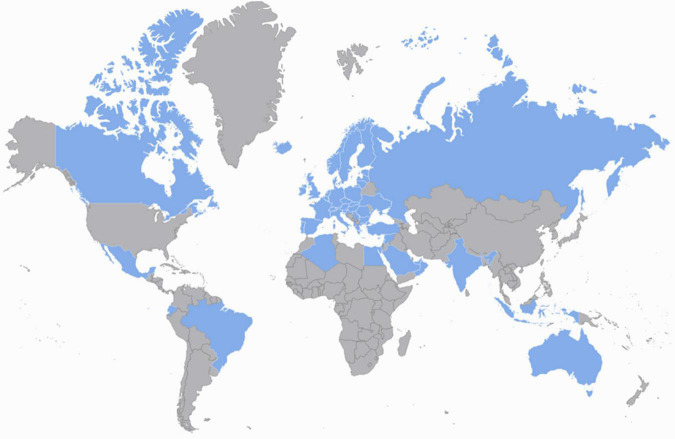
World map showing responding countries (in dark blue): Albania, Algeria, Australia, Austria, Belgium, Brazil, Bulgaria, Canada, Croatia, Cyprus, Czechia, Denmark, Ecuador, Egypt, Estonia, Finland, France, Germany, Greece, Hungary, Iceland, India, Indonesia, Ireland, Israel, Italy, Latvia, Lebanon, Lithuania, Luxembourg, Mexico, Netherlands, Norway, Oman, Poland, Portugal, Romania, Russia, Serbia, Slovakia, Sweden, Saudi Arabia, Slovenia, Spain, Switzerland, Syria, Turkey, Ukraine, United Arab Emirates, United Kingdom.

A hundred and forty-three health care professionals (143) completed the survey representing 122 PICUs from 50 countries, with the most units (98/122) from Europe (Figure 1). Following the removal of duplicates, 122 surveys were analyzed. As we asked our contacts to disseminate the survey amongst their respective country networks, thus without a known denominator, we are unable to calculate a precise response rate. Most of the respondents were physicians (*N* = 107/122, 88%), and among these, most (75% 81/107) were pediatricians and 24/122 (20%) were anesthetists working in PICU. Nine nurses (7%) and six (5%) dieticians completed the survey. Among the 122 responding PICUs, 64 (52%) were general, 36 (30%) were mixed general-cardiac and six (5%) were standalone cardiac PICUs. Fourteen (11%) units were mixed PICU–NICUs, two (2%) were mixed pediatric and adult ICUs.

### Fasting Times Prior to Extubation

**FIGURE 2:**
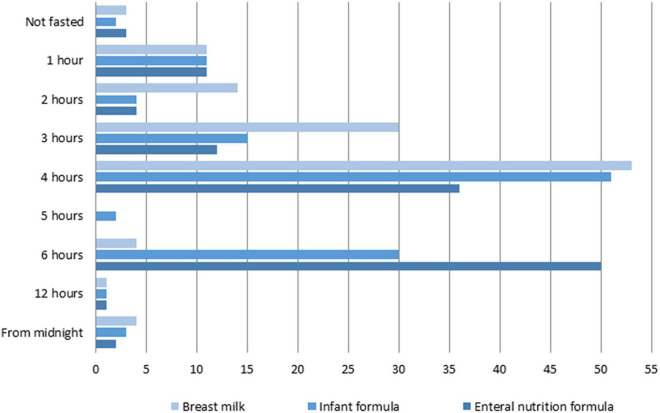
Number of PICUs showing various lengths of fasting depending on feed type.

The duration of fasting varied between PICUs and depended on feed type (Figure 2). Forty percent of units fasted infants routinely for 4 h, irrespectively of feed type. A quarter (25%) fasted infants receiving breast milk for 3 h, and formula-fed infants for 6 h (Figure 2). Older children were most often fasted for six (41%) and 4 h (30%).

### Fasting Guidelines

Half (61/122, 50%) of respondents stated their units had a written protocol for fasting prior to extubation, while 55/122 (45%) did not and 4% did not know. Nine respondents from six countries confirmed the presence of a national guidance on pre-extubation fasting (Bahrain, Netherlands, Oman, Serbia, Turkey, United Kingdom). The majority, 77 (63%) stated there was no national guidance, with 28 (23%) unaware of any. Around half (60/122, 49%) of respondents reported their local pre-extubation fasting practices were based on national guidance for fasting prior to elective anesthesia in children, with 40% (49/122) not and 10% (12/122) unsure.

### Fasting Practices Prior to Extubation

Fasting practices prior to extubation varied depending on specific patient circumstances. Fasting was commonly used in patients receiving gastric feeds, those diagnosed with gastro-esophageal reflux and when the perceived risk for re-intubation was high, or use of non-invasive ventilation (NIV) was planned. Patients receiving jejunal feeds were fasted less often (Table 1).

**TABLE 1:** Fasting prior to extubation in PICU specific circumstances.

	Always	Often	Sometimes	Never
Low risk extubation, gastric feeding (*N* = 122)	86 (70%)	21 (17%)	10 (8%)	5 (4%)
Low risk extubation, jejunal feeding (*N* = 121)	46 (38%)	23 (19%)	35 (29%)	17 (14%)
High risk extubation (*N* = 122)	102 (84%)	12 (10%)	7 (6%)	1 (0.8%)
Extubation to NIV (*N* = 122)	85 (70%)	19 (15%)	14 (11%)	4 (3%)
Known gastro-esophageal reflux (*N* = 121)	83 (68%)	18 (15%)	20 (16%)	0

### Aspiration of the Gastric Feeding Tube Prior to Extubation

Most units stated they aspirated the gastric tube (GT) and performed it immediately prior to extubation [always: 55 (45%), often: 20 (16%), sometimes 26 (21%) and never 12 (10%)] rather than at the time of commencement of fasting [always: 26 (21%), often: 21 (17%), sometimes 26 (21%) and never 39 (32%)].

### Re-introducing Feeds Following Extubation

Enteral (gastric/jejunal) feeds were re-introduced sooner after extubation than oral feeds. Seventy eight percent of PICUs (78%, *N* = 96) re-introduced enteral feeding within 5 h after extubation, while 62% (*N* = 76) re-introduced oral feeding (including breast milk) within this time (Table 2).

**TABLE 2:** Oral/Enteral feeding re-introduction after extubation in PICUs.

	Oral feeds (Breast milk, bottle, food)	Enteral feeds (gastric/jejunal)
When the child wants	45 (37%)	12 (10%)
If the child looks well	45 (37%)	55 (45%)
After first satisfactory blood gas	13 (11%)	19 (16%)
After 1 h	17 (14%)	30 (25%)
After 2 h	23 (19%)	34 (28%)
After 3 h	11 (9%)	17 (14%)
After 4 h	20 (16%)	31 (25%)
After 5 h	6 (5%)	10 (8%)
After 6 h	12 (10%)	7 (6%)
After more than 6 h	14 (11%)	7 (6%)
The following day	14 (11%)	2 (2%)

*Cumulatively, oral and gastric/jejunal nutrition were re-introduced after more than 5 h in 46 (38%) and 26 (21%) of PICUs respectively.*

### Rationales Behind the Pre-extubation Fasting Practice

Results are presented in Tables 3, 4. The risk of vomiting/aspiration and reducing nutritional deficit were the main reasons for fasting children [78/122 (64%)] or reducing fasting times [57/122 (47%)] respectively.

**TABLE 3:** Estimated rates of extubation failure and use of non-invasive ventilation (NIV).

Estimated rates of extubation failure	Number of PICUs	Estimated rates of NIV use	Number of PICUs
≤ 3%	50 (41%)	≤ 3%	12 (10%)
4–5%	25 (20%)	4–5%	18 (15%)
6–10%	11 (9%)	6–10%	25 (20%)
11–25%	4 (3%)	11–25%	19 (16%)
25–50%	0	25–50%	10 (8%)
>50%	1 (1%)	> 50%	5 (4%)
Don’t know	23 (19%)	Don’t know	25 (20%)
No answer	8 (7%)	No answer	8 (7%)

**TABLE 4:** Proposed rationales for fasting and not fasting practices.

Rationale supporting fasting before extubation	Number of PICUs
Potential need for re-intubation	89 (73%)
Risk of regurgitation/aspiration after extubation	78 (64%)
Risk of regurgitation/aspiration prior to extubation	32 (26%)
Pre-extubation fasting is mandatory in my unit	22 (18%)
Fasting before extubation is not practiced in my unit	5 (4%)
Rationale supporting not fasting before extubation	
To maximize nutrition delivery	57 (47%)
To avoid hypoglycemia	29 (24%)
Low risk of peri-extubation aspiration	50 (41%)
Low incidence of aspiration	49 (40%)
Low incidence of re-intubation	47 (39%)
Rapid sequence intubation can be performed in case of re-intubation	43 (35%)
Avoiding delay in extubation	48 (39%)
No valid rationale	16 (13%)

### Ultrasound Techniques to Assess Gastric Emptiness

Half of respondents (61/122, 50%) were aware of gastric POCUS as a technique to assess gastric volume. However, only five PICUs (4%) reported the technique was used.

## Discussion

This is the first survey to explore PICU practices around peri-extubation fasting internationally. As anticipated, we found large variability across PICUs in fasting practices both before and after extubation and limited use of newer gastric POCUS technique to measure stomach volume, with an over reliance on prolonged fasting times to ensure an “empty stomach” at extubation.

Early EN is associated with improved clinical outcomes in critically ill children (3, 4, 11, 12). Inadequate delivery of EN has been associated with poor clinical outcomes such as worsening of underlying malnutrition, longer mechanical ventilation days, longer PICU stay, multi-organ dysfunction and increased mortality (3, 4, 13). Over the last decade efforts have been made to conduct research and produce evidence-based guidelines to improve clinical outcomes through optimizing the EN (stepwise algorithms, volume-based and/or jejunal feeding, etc.) (1, 2). Yet there are still considerable challenges resulting in suboptimal delivery of EN in PICUs. Feeding difficulties observed in critically ill children, can be related to dysmotility, delayed gastric emptying and (perceived) feed intolerance (4–6, 14–18). Some arise from the critical illness and pathophysiology itself and others are iatrogenic in nature (14, 15). In addition, frequent periods of peri-procedural (pre- and post-procedural) fasting commonly occur (4, 5) adding to the cumulative macronutrient deficit (4, 13). The duration of fasting has been shown to be a predictor of the inadequacy of EN delivery (4). A single center study reported that fasting for procedures occurred in 43% children in one 24-h period, with a mean fasting time of 8.9 h (19). Fasting for extubation accounted for 24% of the EN interruptions, in a study where patients spent on average 40% of their PICU length of stay without EN (5). This is consistent with other pediatric (20) and adult ICU studies (21, 22). In adults, airway management procedures caused approximately one-third of the energy deficit observed in critically ill patients (22, 23). A significant proportion of EN interruptions may be avoidable (5, 17) and the need for peri extubation fasting should be rationalized. In our survey, most responders were concerned about the impact of peri-extubation fasting on nutritional deficit.

There is little evidence to guide clinicians about which procedures or therapies critically ill children should be fasted for and for how long. Based on the extrapolation of pre-elective anesthesia fasting recommendations (8, 9), fasting critically ill children prior to extubation, assumes that an empty stomach may help minimize the risk of gastric contents aspiration before, during and after extubation, as shown by our survey results. However, the extrapolation of these guidelines to PICU needs to be challenged as they rely on studies conducted in healthy children (eating a full, normal diet), before elective surgery or procedure. No study has yet been conducted in critically ill children, who may present with different gastric emptying patterns and who are not eating a normal diet. Gastroparesis is common in critically ill children and multifactorial (14, 15); as a result, gastric emptying may be delayed. Continuous enteral feeding is commonly used in PICU and may require shorter (or longer) times for the stomach to empty compared to healthy children’s diet. Consequently, pre-elective anesthesia fasting times may not guarantee an empty stomach in the critically ill child and their use in PICU may lead to unnecessary prolonged fasting times, and delay extubation, and further increase any energy/protein debts. In adults, fasting guidelines adequately predicted gastric emptying only in healthy patients (24). In addition, one study conducted in critically ill adults recently assessed gastric volume with POCUS and found that fasting duration did not correlate with gastric volume and emptiness at extubation (25).

The feared risk of aspiration persists around the time of extubation in critically ill children when oropharyngeal dysfunction and suppression of the airway protection reflexes may persist after removal of the endotracheal tube. In fact, as children are recovering from a period of critical illness, their neurological status may remain altered, due to the effects of opiate and sedative drugs, iatrogenic withdrawal syndrome or primary neurological pathology. Vomiting can occur in this situation (and may be triggered by airway suctioning prior to extubation) which may lead to pulmonary aspiration and pneumonia. The need for re-intubation must also be considered, regardless of how low this might be. Re-intubation has been identified as a risk factor for developing pediatric ventilator associated pneumonia, likely secondary to aspiration (26) and in fact it was the highest rated rationale supporting pre-extubation fasting in our survey (73% of respondents).

In this survey, most units reported routinely stopping EN prior to extubation of low-risk patients, with few reporting not fasting at all for these; half of respondents stated their local pre-extubation practices were based on the guideline for fasting prior to the elective anesthesia, yet their practices varied. Guidance for fasting times prior to elective anesthesia were updated in 2022 with shorter fasting times (8) but delays in implementation of these new recommendations might explain these differences.

Fasting was not only undertaken prior to extubation, but also after extubation and times to reintroducing enteral and oral feeding were considerable in some units. A third of PICUs delayed re-introducing oral feeds for over 6 h after extubation, with 11% withholding oral feeds until the following day, while just under half (37%) of units re-commenced oral feeds based on child demand. The latter approach is consistent with the newest anesthetic guidance advocating a more liberal approach to post elective anesthesia withholding of oral intake (8).

Bedside gastric POCUS has been recognized as a potential tool for assessing gastric volume in children undergoing elective surgery and is recommended in the latest European guidelines (2022) on pre-operative fasting in case of emergency surgery or when fasting instructions have not been applied (8, 9, 25). Spencer et al. have compared gastric content assessed by gastric POCUS and gastric endoscopy and found good correlation (27). Similarly, gastric POCUS could be utilized in PICU to help assess the child’s gastric volume (if the aim is to have an empty or near empty stomach at extubation), potentially shortening the fasting times and accelerating the extubation process. Our survey showed limited use of this technique in practice, despite the increasing awareness.

Despite the concerns around peri-extubation vomiting and aspiration, no association between fasting before elective anesthesia and aspiration has been found, either in adults (28) or children (29). Indeed, in our survey, 40% of respondents perceived a very low risk of peri-extubation aspiration, and some authors suggesting that the risk of aspiration may be over emphasized compared to the risk of malnutrition (30).

Our survey has several limitations that warrant mentioning. Firstly, by its self-report design the survey may reflect individual not unit practices and not real practice. Secondly, we had an overrepresentation of European units, unsurprisingly given our distribution method through the ESPNIC database and members, which means results overall may not reflect non-European centers. Thirdly, some clinical conditions or feeding practice, that may influence PICU health care professional decisions, were not assessed in the survey, such as feeding mode (continuous versus bolus) or constipation. Despite these, we had a large number of responses (122 PICUs) across many countries (50) and we achieved our aim to describe current practices, with this, the first survey to specifically explore the issue of peri-extubation in the PICUs.

## Conclusion

Our international survey showed a large variability in peri-extubation practices in critically ill children on PICU. The extrapolation of pre-elective anesthesia guidelines to the PICU setting may not be beneficial and can mislead clinician decisions. Future studies would help by better describing gastric emptying/gastric volume in PICU patients and modifying fasting times accordingly. Gastric POCUS could provide useful information for the assessment of gastric volume and needs further study in this population.

## Data Availability Statement

The raw data supporting the conclusions of this article will be made available by the authors, without undue reservation.

## Ethics Statement

The studies involving human participants were reviewed and approved by French Group for Pediatric Hepatology, Gastroenterology and Nutrition. Written informed consent for participation was not required for this study in accordance with the national legislation and the institutional requirements.

## Author Contributions

FV, LT, EC, and TN designed the study. FV, TN, LT, FB, and CM disseminated the survey. TN, LT, FV, EC, CM, FB, and LB analyzed the results. TN wrote the draft manuscript. LT English edited the manuscript. All authors reviewed and approved the manuscript.

## Conflict of Interest

The authors declare that the research was conducted in the absence of any commercial or financial relationships that could be construed as a potential conflict of interest.

## Publisher’s Note

All claims expressed in this article are solely those of the authors and do not necessarily represent those of their affiliated organizations, or those of the publisher, the editors and the reviewers. Any product that may be evaluated in this article, or claim that may be made by its manufacturer, is not guaranteed or endorsed by the publisher.
