# Supplementary material for: Planned Peri-Extubation Fasting in Critically Ill Children: An International Survey of Practice
Source: Front Pediatr. 2022 May 11;10:905058. doi: 10.3389/fped.2022.905058 (PMC9132478; doi:10.3389/fped.2022.905058)
Supplement: Supplementary file 1 [file Data_Sheet_1.PDF]

Pre-extubation fasting in PICU

Pre-extubation fasting in critically ill children: European survey of practice

This survey seeks to explore intensive care practices around enteral nutrition management prior to extubation in critically ill children (0 – 17 years old) and their rationale.

Preterms are outside the scope of this survey

We expect only one answer per PICU
The survey may be answered by experienced nurses or consultants working in PICU

By fasting, we mean "interruption of enteral nutrition"

(This survey does not deal with pre-operative fasting)

Your participation in this survey implies your consent that the responses can be used for research. Ethical clearance has been obtained for this study: 30/11/2021 - Ethics committee of the GFHGNP (French Group of Pediatric Hepatology, Gastroenterology & Nutrition) - ref number 2021-036

1. Are you:

- ◯ A pediatrician working in PiCU
- ◯ An anesthetist working in PiCU
- ◯ A Nurse / Advanced nurse practitioner working in pediatric intensive care
- ◯ A dietician working in pediatric intensive care
- ◯ Other (please specify)

2. Where do you work?

Name of the town/City

Name of the PICU (if more than one in this city)

3. In what country do you work?

4. What type of PICU best describes your unit

- General PICU
- Cardiac PICU
- Mixed general and cardiac PICU
- Mixed NICU-PICU
- Mixed PICU-adult ICU
- Other specialist ICU (e.g. neuro, burns)

5. In your PICU, to what extent are children fasted prior to planned extubation in the following circumstances?

	Always	Often	Sometimes	Never
Low risk extubation with gastric feeding	◯	◯	◯	◯
Low risk extubation with post pyloric feeding (jejunal)	◯	◯	◯	◯
Perceived higher risk of extubation failure / re-intubation	◯	◯	◯	◯
Planned NIV post extubation	◯	◯	◯	◯
Infants with known gastro oesophagal reflux	◯	◯	◯	◯

Pre-extubation fasting in PICU

Pre-extubation practice rational

6. Does your unit have a protocol/guideline for extubation that requires fasting?

- Yes
- No
- I don't know

7. Are your unit practices on pre-extubation fasting based on guidance for <u>elective surgery/intubation fasting</u> in healthy children

- Yes
- No
- I don't know

8. In your unit how long are children usually fasted prior to extubation?

	They are not fasted	1h	2h	3h	4h	5h	6h	8h	12h	From midnight
Breast milk / Expressed milk										
Infant formula										
Enteral nutrition formula										

Pre-extubation fasting in PICU

Final questions

9. What would be the rationale for fasting children prior to planned extubation on the PICU?

- [ ] NA: Pre-extubation fasting does not belong to my unit practice
- [ ] It is mandatory (legally or hospital protocol)
- [ ] In case of the need for re-intubation
- [ ] Because of the risk of regurgitation/aspiration prior to extubation
- [ ] Because of the risk of regurgitation/aspiration after extubation
- [ ] Other (please specify)
- [ ] None of the above

10. What could be the rationale for not fasting children prior to planned extubation on the PICU?

- [ ] Aspiration risk is low
- [ ] The incidence of aspiration is rare
- [ ] If reintubation is required, we can use rapid sequence induction
- [ ] The incidence of reintubation is rare
- [ ] To avoid hypoglycaemia
- [ ] To maximise nutritional delivery
- [ ] To avoid delaying extubation
- [ ] Other (please specify)
- [ ] None of the above

11. Are national guidance available in your country on pre-extubation fasting in PICU?

- ( ) Yes
- ( ) No
- ( ) I don't know

12. Do you aspirate the feeding tube to empty the stomach prior to extubation?

	Always	Often	Sometimes	Never
When I stop enteral nutrition administration	◯	◯	◯	◯
Immediately pre extubation	◯	◯	◯	◯

13. In your unit, do you know how many children fail extubation and require reintubation within a few hours?

- ◯ I don t know
- ◯ <1%
- ◯ 2-3%
- ◯ 4-5%
- ◯ 6-10%
- ◯ 11-25%
- ◯ 26-50%
- ◯ >50%

14. In your unit, do you know how many children require non-invasive ventilation (excluding high flow nasal canula) after extubation?

- ◯ I don t know
- ◯ <1%
- ◯ 2-3%
- ◯ 4-5%
- ◯ 6-10%
- ◯ 11-25%
- ◯ 26-50%
- ◯ >50%

15. After extubation, if the child has no sign of clinical deterioration, when would you start enteral nutrition again?

	Oral feeds (Breast milk, bottle, food)	Enteral feeds (eg. nasogastric, gastrostomy)
When the child wants	☐	☐
If the child looks good	☐	☐
after the first blood gas is satisfactory	☐	☐
After 1h	☐	☐
After 2h	☐	☐
After 3h	☐	☐
After 4h	☐	☐
After 5h	☐	☐
After 6h	☐	☐
After more than > 6 hours	☐	☐
The following day	☐	☐

16. Are you aware of ultrasound techniques to assess stomach volume / emptiness (gastric antral ultrasounding)?

- ◯ Yes, we use it
- ◯ Yes, but we do not use it
- ◯ No

17. Free comments
